# Supplementary material for: Pneumatosis Intestinalis Induced by Anticancer Treatment: A Systematic Review
Source: Cancers (Basel). 2022 Mar 25;14(7):1666. doi: 10.3390/cancers14071666 (PMC8996919; doi:10.3390/cancers14071666)
Supplement: Supplementary file 1 [file cancers-14-01666-s001.zip › cancers-1619693-supplementary.pdf]

Supplementary material S1. Search strings

First research

PubMed = 122 results (30/09/2021)
("pneumatosis intestinalis" OR "pneumatosis coli") AND (" adverse event" OR "TKI" OR "tyrosine kinase inhibitor" OR "targeted therapy" OR "anti-angiogenic" OR "immunotherapy" OR "immune check point inhibitor" OR "chemotherapy" OR "solid tumor" OR "cancer" OR "tumor" OR "oncology")

Embase = 72 results (30/09/2021)
('solid malignant neoplasm'/exp OR 'malignant neoplasm, solid' OR 'solid malignant neoplasm' OR 'solid malignant tumor' OR 'solid malignant tumour' OR 'solid tumor' OR 'solid tumour') AND ('targeted therapy'/exp OR 'chemotherapy'/exp OR 'chemotherapeutics' OR 'chemotherapy') AND ('pneumatosis intestinalis'/exp OR 'abdominal gas cyst' OR 'colon air insufflation' OR 'colon pneumatosis' OR 'cystic pneumatosis, intestinal' OR 'emphysema intestinalis' OR 'entero-peritoneal pneumatosis' OR 'enteroperitoneal pneumatosis' OR 'gas cyst, abdominal' OR 'gastrointestinal gas' OR 'gastrointestinal tract gas' OR 'intestinal cystic pneumatosis' OR 'intestinal emphysema' OR 'intestinal intramural gas' OR 'intestinal pneumatosis' OR 'intestinal pneumatosis cystoides' OR 'intestine emphysema' OR 'intestine intramural gas' OR 'intestine pneumatosis' OR 'intramural bowel gas' OR 'pneumatosis coli' OR 'pneumatosis cystoides' OR 'pneumatosis cystoides intestinalis' OR 'pneumatosis intestinalis' OR 'pneumatosis intestini' OR 'pneumatosis, intestinal' OR 'pneumatosis, intestinal cystic' OR 'pneumatosis, intestine' OR 'sigmoid pneumatosis')

Web of Science = 115 results (30/09/2021)
("pneumatosis intestinalis" OR "pneumatosis coli") AND (" adverse event" OR "TKI" OR "tyrosine kinase inhibitor" OR "targeted therapy" OR "anti-angiogenic" OR "immunotherapy" OR "immune check point inhibitor" OR "chemotherapy" OR "solid tumor" OR "cancer" OR "tumor" OR "oncology")

Second research

PubMed = 16 results (07/09/2021)
("Molecular Targeted Therapy"[Mesh] OR "Molecular Targeted Therapies" OR "Targeted Therapy" OR "Molecular Therapy" OR "Molecular Targeted" OR "Targeted Molecular Therapy" OR "Molecular Therapy" OR "Targeted" OR "Targeted Molecular Therapies" OR "Therapy, Targeted Molecular") AND ("Pneumatosis Cystoides Intestinalis"[Mesh] OR "Intestinalis, Pneumatosis Cystoides" OR “pneumatosis intestinalis” OR “pneumatosis cystoides intestinalis” OR “intramural bowel gas” OR “pneumatosis coli”)

Embase = 25 results (03/09/2021)
('molecularly targeted therapy'/exp OR 'molecular target therapy' OR 'molecular targeted therapy' OR 'molecularly targeted therapy' OR 'targeted cancer therapy' OR 'targeted molecular therapy' OR 'targeted therapy (cancer)') AND 'pneumatosis intestinalis'/exp

Web of Science = 30 results (07/09/2021)
(ALL=(pneumatosis) OR ALL=(Pneumatosis intestinalis) OR ALL=(Intramural bowel gas)) AND (ALL=(target therapy) OR ALL=(Tyrosine kinase inhibitor) OR ALL=(immunotherapy))

Table S1. Included papers

Citation number	Paper reference
71.	Adachi K, Okamoto S. An unusual bowel complication during molecularly-targeted therapy. Dig Liver Dis. 2015 Nov;47(11):e20.
67.	Akhtar K, Zayac A, Lemke S. Intestinal Pneumatosis: A Manifestation of Rarely Reported Axitinib-Associated Necrotizing Enterocolitis. Am J Ther. 2018 Dec;25(6):e763–5.
43.	Alfaro MP, Cantera JE, Pérez IV. Portal venous gas secondary to molecular targeted therapy. AJR Am J Roentgenol. 2013 Aug;201(2):W368-369.
68.	Alonso-Burgos A, Nogueral JJ, Cosín O, Viudez A, Pueyo J, Elorz M, et al. [Intestinal pneumatosis and portomesenteric gas: a case review]. Rev Med Univ Navarra. 2007 Jun;51(2):3–6.
69.	Asahi Y, Suzuki T, Sawada A, Kina M, Takada J, Gotoda H, et al. Pneumatosis Cystoides Intestinalis Secondary to Sunitinib Treatment for Gastrointestinal Stromal Tumor. Case Rep Gastroenterol. 2018 Aug;12(2):432–8.
9.	Asmis TR, Chung KY, Teitcher JB, Kelsen DP, Shah MA. Pneumatosis intestinalis: a variant of bevacizumab related perforation possibly associated with chemotherapy related GI toxicity. Invest New Drugs. 2008 Feb;26(1):95–6.
64.	Brocchi S, Parmeggiani A, Gaudiano C, Balacchi C, Renzulli M, Brandi N, et al. Pneumatosis intestinalis and spontaneous perforation associated with drug toxicity in oncologic patients: a case series. Acta Gastroenterol Belg. 2021 Sep;84(3):497–9.
70.	Candelaria M, Bourlon-Cuellar R, Zubieta JLG-L, Noel-Ettiene LM, Sánchez-Sánchez JM. Gastrointestinal pneumatosis after docetaxel chemotherapy. J Clin Gastroenterol. 2002 Apr;34(4):444–5.
72.	Chang C-J, Shen C-I, Wu C-L, Chiu C-H. Pneumatosis intestinalis induced by targeted therapy. Postgrad Med J. 2020 Nov 24;postgradmedj-2020-139232.
62.	Chaudhry NS, Bi WL, Gupta S, Keraliya A, Shimizu N, Chiocca EA. Pneumatosis Intestinalis After Molecular-Targeted Therapy. World Neurosurg. 2019 May;125:312–5.
73.	Clemente G, Chiarla C, Giovannini I, De Rose AM, Astone A, Barone C, et al. Gas in portal circulation and pneumatosis cystoides intestinalis during chemotherapy for advanced rectal cancer. Curr Med Res Opin. 2010 Mar;26(3):707–11.
74.	Coba G, Shahin AV, Saba M, Greene J. Management of Pneumatosis Intestinalis in Neutropenic Acute Leukemia Patients. Infectious Diseases in Clinical Practice. 2019;
10.	Coriat R, Ropert S, Mir O, Billemont B, Chaussade S, Massault P-P, et al. Pneumatosis intestinalis associated with treatment of cancer patients with the vascular growth factor receptor tyrosine kinase inhibitors sorafenib and sunitinib. Invest New Drugs. 2011 Oct 1;29(5):1090–3.
11.	Dashwood AM, Mason R, Jennings C, Dhillon P. Hepatic portal venous gas with associated bowel ischaemia and intra-abdominal sepsis after recent chemotherapy. BMJ Case Rep. 2016 Jan 8;2016:bcr2015213564.
12.	de la Serna S, Luna A, de la Rosa H. Intestinal pneumatosis and pneumoperitoneum in an oncological scenario: a change of attitude. Rev Esp Enferm Dig. 2018 Jan;110(1):68–9.
13.	Di Pietropaolo M, Trinci M, Giangregorio C, Galluzzo M, Miele V. Pneumatosis cystoides intestinalis: case report and review of literature. Clin J Gastroenterol. 2020 Feb;13(1):31–6.
14.	Faria LDBB, Anjos CHD, Fernandes GDS, Carvalho IF da S. Pneumatosis intestinalis after etoposide-based chemotherapy in a patient with metastatic small cell lung cancer: successful conservative management of a rare condition. Einstein (Sao Paulo). 2016 Sep;14(3):420–2.

15. Flaig TW, Kim FJ, La Rosa FG, Breaker K, Schoen J, Russ PD. Colonic pneumatosis and intestinal perforations with sunitinib treatment for renal cell carcinoma. Invest New Drugs. 2009 Feb;27(1):83–7.
16. Fujimi A, Sakamoto H, Kanisawa Y, Minami S, Nagamachi Y, Yamauchi N, et al. Pneumatosis intestinalis during chemotherapy with nilotinib in a patient with chronic myeloid leukemia who tested positive for anti-topoisomerase I antibodies. Clin J Gastroenterol. 2016 Dec;9(6):358–64.
17. Galm O, Fabry U, Adam G, Osieka R. Pneumatosis intestinalis following cytotoxic or immunosuppressive treatment. Digestion. 2001;64(2):128–32.
18. Guiu S, Ortega-Deballon P, Guiu B. Pneumatosis intestinalis and pneumoperitoneum during treatment by paclitaxel. Surgery. 2011 Feb;149(2):297–8.
19. Hashimoto S, Saitoh H, Wada K, Kobayashi T, Furushima H, Kawai H, et al. Pneumatosis cystoides intestinalis after chemotherapy for hematological malignancies: report of 4 cases. Intern Med. 1995 Mar;34(3):212–5.
20. Huang Y-H, Siao F-Y, Yen H-H. Abdominal Distension in a Patient With Hepatocellular Carcinoma. Gastroenterology. 2015 Aug 1;149(2):e12–3.
21. Iwasaku M, Yoshioka H, Korogi Y, Kunimasa K, Nishiyama A, Nagai H, et al. Pneumatosis cystoides intestinalis after gefitinib therapy for pulmonary adenocarcinoma. J Thorac Oncol. 2012 Jan;7(1):257.
22. Jarkowski A, Hare R, Francescutti V, Wilkinson N, Khushalani N. Case report of pneumatosis intestinalis secondary to sunitinib treatment for refractory gastrointestinal stromal tumor. Anticancer Res. 2011 Oct;31(10):3429–32.
23. Kameda T, Nakano K, Yamazaki M, Koshimizu T, Morita T. Axitinib-induced Pneumatosis Intestinalis and Acute Acalculous Cholecystitis in a Patient With Renal Cell Carcinoma. Urology. 2017 Mar;101:e7–8.
24. Kashima T, Ohno Y, Tachibana M. Pneumatosis intestinalis and hepatic portal venous gas in a patient receiving sorafenib. Int J Urol. 2012 Nov;19(11):1041–2.
25. Khan T, Mujtaba M, Flores MS, Nahum K, Carson MP. A Case of Pneumatosis Intestinalis With Pneumoperitoneum as a Potential Delayed Adverse Effect of Capecitabine. World J Oncol. 2019 Jun;10(3):151–2.
26. Kirmanidis M, Boulas KA, Paraskeva A, Kariotis I, Barettas N, Kariotis S, et al. Extensive colonic pneumatosis in a patient on adjuvant chemotherapy after right colectomy for primary terminal ileum lymphoma: A decision-making process between surgical and non-surgical management. Int J Surg Case Rep. 2018;52:84–8.
27. Kouzu K, Tsujimoto H, Hiraki S, Takahata R, Yaguchi Y, Kumano I, et al. A case of pneumatosis intestinalis during neoadjuvant chemotherapy with cisplatin and 5-fluorouracil for esophageal cancer†. J Surg Case Rep. 2017 Nov;2017(11):rjx227.
28. Kung D, Ruan DT, Chan RK, Ericsson ML, Saund MS. Pneumatosis intestinalis and portal venous gas without bowel ischemia in a patient treated with irinotecan and cisplatin. Dig Dis Sci. 2008 Jan;53(1):217–9.
29. Lee JY, Han H-S, Lim S-N, Shim YK, Choi YH, Lee O-J, et al. Pneumatosis intestinalis and portal venous gas secondary to Gefitinib therapy for lung adenocarcinoma. BMC Cancer. 2012 Mar 10;12:87.
30. Lee YS, Han JJ, Kim S-Y, Maeng CH. Pneumatosis cystoides intestinalis associated with sunitinib and a literature review. BMC Cancer. 2017 Nov 9;17(1):732.
31. Maeda A, Nakata M, Shimizu K, Yukawa T, Saisho S, Okita R. Pneumatosis intestinalis after gefitinib therapy for pulmonary adenocarcinoma: a case report. World J Surg Oncol. 2016 Jun 29;14(1):175.
32. Mais L, Galoo E. Pneumatose kystique colique au cours d’une chimiothérapie palliative pour un cancer colorectal [Pneumatosis cystoides intestinalis during palliative chemotherapy for colorectal cancer]. Rev Med Brux. 2012;33(1):48–50.

33. Martín-Lagos Maldonado A, Lozano Cejudo C, Sáenz Lozano A. A pneumoperitoneum due to intestinal cystic pneumatosis associated with a tyrosine kinase inhibitor. Rev Esp Enferm Dig. 2018 Aug;110(8):531–2.
34. Martín-Soberón MC, Ruiz S, De Velasco G, Yarza R, Carretero A, Castellano D, et al. Pneumatosis intestinalis in a radioactive iodine-refractory metastasic thyroid papillary carcinoma with BRAFV600E mutation treated with dabrafenib-trametinib: a case report. J Med Case Rep. 2021 Mar 2;15(1):109.
36. Miller JA, Ford DJ, Ahmed MS, Loree TR. Two Cases of Pneumatosis Intestinalis during Cetuximab Therapy for Advanced Head and Neck Cancer. Case Rep Oncol Med. 2015;2015:214236.
37. Mimatsu K, Oida T, Kawasaki A, Kano H, Kuboi Y, Aramaki O, et al. Pneumatosis cystoides intestinalis after fluorouracil chemotherapy for rectal cancer. World J Gastroenterol. 2008 May 28;14(20):3273–5.
35. Nadav Michaan SHK, Nadav Michaan SHK. A rare case of pneumatosis intestinalis after gastrocystostomy in an ovarian cancer patient under bevacizumab treatment. European Journal of Gynaecological Oncology. 2019 Feb 10;40(1):157–9.
38. Nukii Y, Miyamoto A, Mochizuki S, Moriguchi S, Takahashi Y, Ogawa K, et al. Pneumatosis intestinalis induced by osimertinib in a patient with lung adenocarcinoma harbouring epidermal growth factor receptor gene mutation with simultaneously detected exon 19 deletion and T790 M point mutation: a case report. BMC Cancer. 2019 Feb 28;19(1):186.
39. Nunomiya K, Inoue S, Sato K, Igarashi A, Yamauchi K, Abe Y, et al. Pneumatosis Intestinalis in Lung Cancer Induced Twice by Different Drugs: Bevacizumab and Pemetrexed. Intern Med. 2021 Jul 1;60(13):2109–13.
40. Ohkuma K, Saraya T, Shimoda M, Takizawa H. A case of pneumatosis cystoides intestinalis. J Gen Fam Med. 2017 Dec;18(6):481–2.
41. Ozturk M, Camlidag I, Nural MS, Ozbalci GS, Bekci T. A rare cause of acute abdomen in the ED: Chemotherapy-induced pneumatosis intestinalis. Turk J Emerg Med. 2017 Dec;17(4):151–3.
42. Pengermä P, Katunin J, Turunen A, Rouvelas I, Palomäki A, Kechagias A. Is surgical exploration mandatory in pneumatosis intestinalis with portomesenteric gas? Lesson learned in a neutropenic patient under chemotherapy. ANZ J Surg. 2021 Jun 25;
44. Petrides C, Kyriakos N, Andreas I, Konstantinos P, Chrysanthos G, Athanasios P, et al. Pneumatosis cystoides intestinalis after cetuximab chemotherapy for squamous cell carcinoma of parotid gland. Case Rep Surg. 2015;2015:530680.
45. Ramos JA, Festic E. Images in clinical medicine. Catastrophic gastroduodenal pneumatosis. N Engl J Med. 2014 Sep 25;371(13):e19.
46. Salcin E, Omercikoglu S, Eroglu SE, Akoglu H, Onur O, Denizbasi A. Does Pneumatosis Intestinalis after Etoposide Chemotherapy always have a Benign Course? J Emerg Med Case Rep. 2016 Mar 21;7(2):24–6.
47. Sassi C, Pasquali M, Facchini G, Bazzocchi A, Battista G. Pneumatosis intestinalis in oncologic patients: when should the radiologist not be afraid? BJR Case Rep. 2017;3(1):20160017.
48. Sherman E, Ramadas P, Lemke S. Cetuximab-Associated Pneumatosis Intestinalis. Am J Ther. 2019 Oct;26(5):e609–10.
49. Shih I-L, Lu Y-S, Wang H-P, Liu K-L. Pneumatosis coli after etoposide chemotherapy for breast cancer. J Clin Oncol. 2007 Apr 20;25(12):1623–5.
50. Shikuma H, Inoue S, Hatayama T, Mukai S, Muto M, Miyamoto S, et al. Pneumatosis cystoides intestinalis linked to sunitinib treatment for renal cell carcinoma. IJU Case Rep. 2019 Nov;2(6):318–20.

51. Shin D-K, Oh J, Yoon H, Kim JE, Chong SY, Oh D. Asymptomatic pneumatosis intestinalis following chemotherapy for B lymphoblastic leukemia with recurrent genetic abnormalities in an adolescent patient. Korean J Hematol. 2012 Mar;47(1):74–6.

52. Thornton E, Howard SA, Jagannathan J, Krajewski KM, Shinagare AB, O'Regan K, et al. Imaging features of bowel toxicities in the setting of molecular targeted therapies in cancer patients. Br J Radiol. 2012 Oct;85(1018):1420–6.

53. Uruga H, Moriguchi S, Takahashi Y, Ogawa K, Murase K, Mochizuki S, et al. Gefitinib successfully administered in a lung cancer patient with leptomeningeal carcinomatosis after erlotinib-induced pneumatosis intestinalis. BMC Cancer. 2018 Aug 16;18(1):825.

54. Vargas A, Pagés M, Buxó E. Pneumatosis intestinalis due to 5-fluorouracil chemotherapy. Gastroenterol Hepatol. 2016 Dec;39(10):672–3.

55. Vijayakanthan N, Dhamanaskar K, Stewart L, Connolly J, Leber B, Walker I, et al. A review of pneumatosis intestinalis in the setting of systemic cancer treatments, including tyrosine kinase inhibitors. Can Assoc Radiol J. 2012 Nov;63(4):312–7.

75. Viswanathan C, Bhosale P, Ganeshan DM, Truong MT, Silverman P, Balachandran A. Imaging of complications of oncological therapy in the gastrointestinal system. Cancer Imaging. 2012 May 7;12:163–72.

76. Viswanathan C, Truong M, Sagebiel T, Garg N, Bhosale P. Imaging of chemotherapy-related iatrogenic abdominal and pelvic conditions. Radiol Clin North Am. 2014 Sep;52(5):1029–40.

77. Yamatoji M, Shiiba M, Yamamoto J, Takahara T, Takeuchi S, Sawai Y, et al. A rare case of pneumatosis intestinalis during adjuvant chemoradiotherapy for oral cancer. Journal of Oral and Maxillofacial Surgery, Medicine, and Pathology. 2021;

65. Yik B, Shah N. Gas Bubbles: A Persistent Problem with Immunotherapy. Dig Dis Sci. 2021 Aug;66(8):2542–4.

78. Yoon S, Hong YS, Park SH, Lee JL, Kim TW. Pneumatosis intestinalis after cetuximab-containing chemotherapy for colorectal cancer. Jpn J Clin Oncol. 2011 Oct;41(10):1225–8

Table S2. Comorbidities

	**Oncological (72 cases)**	**Hematological (16 cases)**	**Total (88 cases)**
**Reported comorbidity - no (%)**	**15 (20.8)**	**3 (18.8)**	**18 (20.5)**
Hypertension	6 (8.3)	1 (6.3)	7 (8.0)
Dislipidemia	4 (5.6)	1 (6.3)	5 (5.7)
Type 1 Diabetes	2 (2.8)	-	2 (2.3)
Type 2 Diabetes	3 (4.2)	-	3 (3.4)
Hypothyroidism	2 (2.8)	-	2 (2.3)
Acute pulmonary thromboembolism	1 (1.4)	-	1 (1.1)
Chronic obstructive pulmonary disease	1 (1.4)	-	1 (1.1)
Idiopathic interstitial pneumonia	-	1 (6.3)	1 (1.1)
Bronchial asthma	1 (1.4)	-	1 (1.1)
Cor pulmonale	1 (1.4)	-	1 (1.1)
Chronic heart failure	1 (1.4)	1 (6.3)	2 (2.3)
Chronic hepatitis B - cirrhosis	1 (1.4)	-	1 (1.1)
B-thalassemia	-	1 (6.3)	1 (1.1)
Anemic syndrome	1 (1.4)	-	1 (1.1)
Rheumatoid arthritis	1 (1.4)	-	1 (1.1)
Solid organ transplant	1 (1.4)	-	1 (1.1)
